# Path planning for UAVs in complex terrain based on the PGD model: Algorithmic improvements combining feature extraction and reinforcement learning

**DOI:** 10.1371/journal.pone.0340394

**Published:** 2026-02-03

**Authors:** Liangshuai Liu, Xiaofeng Li, Lingming Meng, Yuntao Zhao, Yaya Lv

**Affiliations:** State Grid Hebei Electric Power Research Institute, Shijiazhuang, Hebei, China; Universidade Federal de Uberlandia, BRAZIL

## Abstract

This paper proposes the PGD model for UAV path planning in complex terrain, addressing key challenges such as high-dimensional state processing, blind path exploration, and poor cross-scene adaptability. The PGD model integrates Transformer, GAN, and DDPG, forming a “compression-generation-optimization" closed-loop system. The Transformer module compresses high-dimensional terrain data, alleviating training bottlenecks, while the GAN module generates high-quality candidate paths, reducing ineffective exploration. DDPG then optimizes the path planning strategy efficiently. Experimental results demonstrate the superior performance of PGD on the UAVDT (suburban) and AirSim (canyon) datasets. In terms of path length (*P*_*l*_), PGD achieves 20.0m/22.0m, compared to baseline models such as PPO-DRL (23.8m) and Soft Actor-Critic (24.0m). PGD also outperforms in collision rate (*C*_*r*_) with 2.5%/3.0% and computational efficiency (*T*_*c*_) with 13.5s/16.0s, respectively. The PGD model shows significant improvements in path planning efficiency and adaptability, particularly in high-complexity terrains. Compared to traditional models, PGD’s multi-module synergy enhances feature correlation and physical path constraints, offering a novel framework for intelligent planning in complex environments. Future work will focus on enhancing model adaptability to extreme weather and multi-agent collaborative scenarios.

## Introduction

In complex terrain scenarios such as mountain power inspection and canyon geological monitoring, unmanned aerial vehicles (UAVs), due to their flexibility and maneuverability, have become essential tools to replace manual operations in high-risk and low-efficiency environments [1,2]. However, these environments are characterized by rugged terrain structures and irregularly distributed, dynamically changing obstacles (e.g., sudden rockfalls, vegetation growth) [3,4]. This places three core requirements on UAV path planning systems: collision-free path safety, path length efficiency, and real-time decision responsiveness [5,6]. Traditional path planning methods have gradually shown limitations in such environments, with studies identifying their key bottlenecks. Algorithms like A* [7] and Dijkstra [8,9], which are based on graph search, rely on manually designed cost functions. Although they can guarantee optimal solutions in static and structured environments, the balance between actual cost and heuristic estimation in their evaluation functions lacks generality [10–12]. In unstructured terrains, this often leads to paths that either cling too closely to obstacles or become unnecessarily long due to biased weight settings, making it difficult to balance safety and efficiency. Sampling-based methods such as RRT* overcome the dependency of graph search algorithms on environmental modeling by exploring high-dimensional spaces through random sampling [13,14]. However, studies have shown that as terrain complexity increases, the correlation between sampled points and the target region significantly decreases, resulting in an exponential decline in planning efficiency. In constrained spaces such as narrow canyons, dense local sampling often leads to suboptimal solutions [15].

The rise of deep learning has provided a new pathway to overcome these bottlenecks, yet existing techniques still face multidimensional challenges [16,17]. At the feature processing level, studies have confirmed the inherent limitations of different network architectures. CNNs excel at extracting local obstacle features from terrain images but, due to their fixed receptive fields, fail to capture long-range terrain correlations such as “mountain peak–valley” relationships, leading to insufficient perception of global path constraints and discontinuous trajectories in highly undulating mountain scenes [18–20]. RNNs, such as LSTMs, can model temporal sequences to process UAV motion states [21]; however, their gradient transmission properties make it difficult to learn long-term path strategies in highly dynamic terrains, often causing trajectory oscillations during complex turns. Graph Neural Networks (GNNs) can model the topological relationships between obstacles, yet in sparse terrain data (e.g., missing point cloud regions in remote mountains), insufficient node connections cause severe performance degradation, and they cannot directly output continuous action commands to meet UAV flight control requirements [22,23].

Reinforcement learning (RL) has made significant progress in autonomous decision-making. The Deep Deterministic Policy Gradient (DDPG) algorithm [24,25], due to its suitability for continuous action spaces, has become the mainstream framework for path planning. However, in high-dimensional state inputs (such as 3D point clouds and meteorological data that form thousand-dimensional features), DDPG suffers from slow convergence and training instability [26,27]. In complex terrains, it often requires tens of thousands of iterations to generate a feasible path. The collaboration between feature extraction and decision-making modules also faces a gap: Transformer [28–30], with its self-attention mechanism, excels at capturing global dependencies in high-dimensional data but is often treated as an independent preprocessing module, lacking dynamic interaction with subsequent decision-making processes, leading to potential loss of key path information in compressed features.

The path generation stage also presents a contradiction: Generative Adversarial Networks (GANs) [31,32] can generate diverse path solutions, but their generation process lacks sufficient encoding of the drone’s physical constraints (such as maximum climb angle and turning radius), resulting in “theoretically feasible but practically unfeasible" paths (e.g., vertical climb trajectories on steep slopes); Variational Autoencoders (VAE) [33] ensure generation stability through probabilistic modeling but struggle to handle sudden obstacles due to insufficient sample diversity. More critically, existing research often focuses on optimizing individual modules, lacking the end-to-end collaboration of “feature compression - path generation - policy optimization". The disconnection between feature extraction and path generation leads to candidate paths that do not satisfy the core constraints of the terrain, while the separation between path generation and policy optimization causes reinforcement learning to fall into ineffective exploration, preventing the full potential of each module from being realized. Even with the emergence of hybrid or hierarchical reinforcement learning models in recent years (such as PPO with attention mechanism and hybrid graph-RL method), a complete process of “high-dimensional data processing - path generation constraints - continuous action optimization" has not yet been formed. Either the lack of an active path generation mechanism leads to blind exploration, or it is difficult to take into account the global correlation and physical constraints of the terrain, and thus cannot completely solve the core pain points of complex terrain.

To address the aforementioned issues, this paper proposes a Perception-Generation-Decision (PGD) model that integrates feature extraction and reinforcement learning. This model achieves efficient path planning in complex terrain through the organic collaboration of Transformer, GAN, and DDPG. Its core innovations are reflected in three aspects: First, it constructs a dynamic collaborative feature compression mechanism, utilizing Transformer’s self-attention mechanism to focus on the key associations between “UAV-obstacle-inspection point." While compressing high-dimensional terrain data, it retains key decision-making information through dynamic interaction with subsequent modules, alleviating the training pressure of reinforcement learning. Second, it designs a constraint-aware path generation framework, using the simplified features output by Transformer as conditions to guide GAN in generating high-quality path candidates that conform to physical constraints, providing “prior experience" for reinforcement learning and reducing ineffective exploration. Third, it establishes a closed-loop collaborative optimization system, enabling the feature compression results to simultaneously serve path generation constraints and reinforcement learning state inputs. Path candidates serve as “demonstration data" for policy optimization, accelerating convergence and forming a complete, complementary link. This systematically solves the core challenges of UAV path planning in complex terrain, particularly in high-dimensional state processing, blind path exploration, and cross-scene adaptability.

## Materials and methods

### Problem description

To address the core requirement of drone inspection path planning in complex terrains — achieving efficient and safe path decisions in high-dimensional state spaces — this paper abstracts the problem as a constrained continuous state Markov Decision Process (MDP). The focus is on capturing the high-dimensional characteristics of the state space, the continuity of action decisions, and the objective function that aligns with the “Feature Compression - Path Generation - Policy Optimization" framework of the PGD model, providing clear boundaries for the subsequent module design.

The state space is the core input for the model’s perception of the complex terrain, and its high dimensionality is the key bottleneck that leads to the inefficiency of traditional reinforcement learning. Let the state space be *S*, and the state vector s∈S must fully include three categories of information: First, the drone’s own state, including three-dimensional coordinates (*x*, *y*, *z*) (*x*, *y* are horizontal positions, *z* is altitude), heading angle θ, flight speed *v*, and climb rate $\dot{z}$, which directly determine the physical basis for action execution; second, the environmental features of the complex terrain, extracted via LiDAR or 3D point cloud data, represented by the relative coordinates $\left(d_{1}^{x},d_{1}^{y},d_{1}^{z},\ldots ,d_{k}^{x},d_{k}^{y},d_{k}^{z}\right)$ of the *k* closest obstacles to the drone ($d_{i}^{x},d_{i}^{y},d_{i}^{z}$ represent the relative distances of the *i*-th obstacle in the three-dimensional direction). This information is highly unstructured in mountainous and canyon environments and is the main source of state dimension inflation; third, the inspection task information, including the remaining target point coordinates $\left(t_{1}^{x},t_{1}^{y},t_{1}^{z},\ldots ,t_{m}^{x},t_{m}^{y},t_{m}^{z}\right)$ and their respective distances $l_{1},\ldots ,l_{m}$ (*m* is the number of remaining points), ensuring that path planning aligns with the task goals. The specific form of the state vector is:

$$s=\left[x,y,z,\theta ,v,\dot{z},d_{1}^{x},d_{1}^{y},d_{1}^{z},\ldots ,d_{k}^{x},d_{k}^{y},d_{k}^{z},t_{1}^{x},t_{1}^{y},t_{1}^{z},\ldots ,t_{m}^{x},t_{m}^{y},t_{m}^{z},l_{1},\ldots ,l_{m}\right]$$ (1)

In typical complex terrains (such as canyons with multiple obstacles), the increase in *k* and *m* can cause the state dimension to exceed a thousand, which is the core motivation for introducing Transformer for feature compression — by focusing on the key associations between “drone - obstacles - inspection points," the computational burden on subsequent modules is reduced.

The action space needs to match the continuous control characteristics of the drone, while providing output dimensions suitable for the policy optimization of DDPG. Let the action space be *A*, and the action vector a∈A is defined as a combination of three continuous control quantities: speed adjustment Δv, heading angle adjustment Δθ, and climb angle adjustment Δϕ, i.e.:

a=[Δv,Δθ,Δϕ] (2)

Each component must satisfy physical constraints: $\Delta v\in [\Delta v_{\text{min}},\Delta v_{\text{max}}]$ (limiting the speed change range to avoid overloading the power system), $\Delta \theta \in [\Delta \theta_{\text{min}},\Delta \theta_{\text{max}}]$ (limiting the steering range to ensure flight stability), $\Delta \phi \in [\Delta \phi_{\text{min}},\Delta \phi_{\text{max}}]$ (limiting climb/descent angles to accommodate terrain slope changes). These constraints will serve as hard conditions when generating path candidates with GAN, ensuring the generated path segments are physically executable.

The design of the objective function needs to balance the core metrics of path planning while providing a basis for the reinforcement learning reward mechanism. This paper aims to minimize the comprehensive cost *J*, expressed as:

$$J=\min\left(\omega_{1}C_{\text{len}}+\omega_{2}C_{\text{col}}+\omega_{3}C_{\text{task}}+\omega_{4}C_{\text{smooth}}\right)$$ (3)

where $C_{\text{len}}$ is the path length cost (total flight distance), promoting path economy; $C_{\text{col}}$ is the collision cost (increasing sharply when the distance to obstacles is less than a safety threshold ϵ), ensuring safety; $C_{\text{task}}$ is the task cost (the weighted sum of distances to remaining inspection points), ensuring task completion; the newly added $C_{\text{smooth}}$ is the smoothness cost (the change in angle between adjacent actions), reducing unnecessary sharp turns in complex terrains, and this metric aligns with the smoothness constraint of the path generated by GAN. $\omega_{1}\sim \omega_{4}$ are weight coefficients, which can be dynamically adjusted according to the scene (e.g., increasing $\omega_{2}$ and $\omega_{4}$ in canyon scenarios to prioritize obstacle avoidance and smooth flight).

In terms of dynamic constraints, the state transition of the drone must follow physical laws: the position at the next time step $\left(x^{\prime },y^{\prime },z^{\prime }\right)$ is determined by the current state and action, i.e.:

$$x^{\prime }=x+v\cdot \cos\theta \cdot \Delta t,\;y^{\prime }=y+v\cdot \sin\theta \cdot \Delta t,\;z^{\prime }=z+v\cdot \sin\phi \cdot \Delta t$$ (4)

where Δt is the sampling interval. This transition rule will serve as the foundation for Transformer to extract temporal features, while providing real environmental feedback for the policy evaluation of DDPG.

### Datasets

To thoroughly validate the performance of the PGD model in path planning for complex terrains, this paper selects two representative public datasets: the AirSim Dataset (https://universe.roboflow.com/airsim-gate/airsim-drone-gate) and the UAVDT Dataset (https://opendatalab.com/OpenDataLab/UAVDT). The selection criteria are based on three dimensions: first, the complexity of the terrain, which should include features such as mountains and canyons that match the research scenario; second, data completeness, which should provide full-dimensional information such as drone states, environmental features, and trajectory annotations to support model training; third, scene authenticity, balancing both simulator-generated data and real-world data to validate the model’s generalization ability.

The AirSim Dataset [34] is a drone simulation dataset developed by Microsoft Research, with its core advantage being the ability to customize complex terrain environments, along with comprehensive data dimensions. The dataset is built on the Unreal Engine and includes various pre-configured complex terrain scenarios (such as mountains, canyons, and urban-rural junctions). For this study, the “Canyon Mountain" subscene is selected, which simulates rugged terrain with an elevation difference of over 500 meters, featuring natural obstacles such as rocks and trees, as well as man-made inspection targets such as transmission towers, closely matching the “complex terrain inspection" scenario studied here. Each sample in the dataset contains the drone’s six degrees of freedom state (position, attitude, speed), LiDAR point cloud data (used to extract obstacle features), high-definition RGB images (used for visual-assisted positioning), and manually annotated optimal inspection trajectories (used as reference benchmarks).

The UAVDT Dataset [35] is a real-world drone video and trajectory dataset, primarily collected from suburban and urban-rural junction scenes, including flight data under different weather conditions such as sunny and cloudy days. The core value of this dataset lies in its provision of real terrain physical characteristics (such as actual obstacle distribution and terrain undulation patterns), which can effectively validate the model’s adaptability in non-simulation environments. The dataset includes GPS trajectories of the drone (converted from latitude and longitude to 3D coordinates), flight speed and heading angle records, ground-truth obstacle annotations (such as the position and height of houses and trees), and corresponding aerial image sequences for each scene.

A comparison of the key information from both datasets is shown in Table 1. By training the model on the AirSim Dataset, the controllable variables of complex terrain (such as obstacle density and terrain slope) can be fully utilized to validate the core improvements of the PGD model; testing the model on the UAVDT Dataset enables the evaluation of the model’s generalization performance in real-world scenarios. The combination of both datasets provides a complete experimental data support system.

**Table 1:** Core features and experimental applications of the AirSim and UAVDT datasets.

Dataset	Scene Type	Terrain Complexity	Data Content	Sample Size	Purpose
AirSim	Simulator Generated (Canyon Mountain)	High (Elevation difference > 500m, Dense obstacles)	6 DOF state, LiDAR point cloud, RGB images, Annotated trajectories	1000+ trajectories (each with 500+ state frames)	Model Training and Performance Optimization
UAVDT	Real World (Suburban / Urban-Rural Junction)	Medium-High (Elevation difference 100-300m, Scattered obstacles)	GPS trajectory, speed/heading, obstacle annotations, Aerial images	300+ trajectories (each with 300+ state frames)	Model Testing and Generalization Verification

Note: In the table, “Sample Size" refers to the number of valid trajectories used in the experiment, and the number of state frames per trajectory.

Regarding data accuracy, the terrain simulation parameters and UAV physical models in the AirSim Dataset are calibrated with real flight data, with a deviation of ≤5% between simulation and actual trajectories. The UAVDT Dataset corrects GPS data through differential positioning, verifies obstacle annotations using LiDAR and manual review, and ensures static obstacle position errors of < 0.5 meters and height errors of < 0.3 meters, guaranteeing data reliability. In terms of information completeness, AirSim includes meteorological interference data, and UAVDT includes multi-time period illumination and vegetation change samples, both covering the core dimensions of UAV status and environmental characteristics required for the entire model training and testing process, without the need for additional data. In addition, neither of these datasets has limitations in terms of generalization ability.

### PGD model architecture

**Fig 1:**
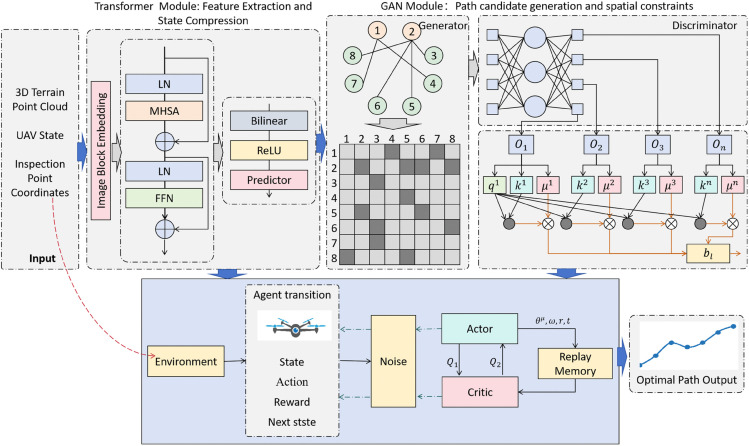
The overall architecture of the PGD model: The raw input data (such as 3D terrain point clouds, drone states, and inspection point coordinates) undergoes feature compression, path generation, and policy optimization to output the globally optimal path that satisfies safety, efficiency, and task requirements.

The PGD (Perception-Generation-Decision) model constructs a complete solution for drone inspection path planning in complex terrains with a “Feature Compression - Path Generation - Policy Optimization" collaborative logic. Its core lies in the organic linkage of three modules, transforming high-dimensional terrain data into features that can be efficiently processed, generating constraint-compliant path candidates, and ultimately optimizing the global optimal path. The overall framework is shown in Fig 1.

**Fig 2:**
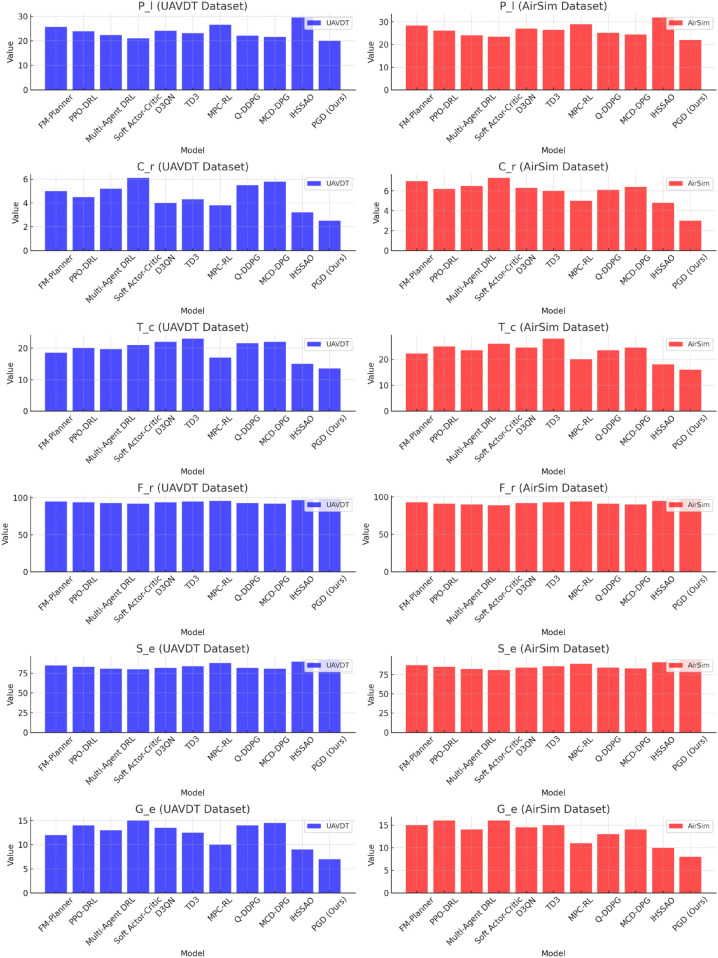
Comparison of UAV path planning models under the UAVDT and AirSim datasets in terms of *P*_*l*_, *C*_*r*_, *T*_*c*_, *F*_*r*_, *S*_*e*_, and *G*_*e*_ metrics.

From the data flow in the model diagram (Fig 2), we can see that the raw input (such as 3D terrain point clouds, drone states, and inspection point coordinates) first enters the Transformer module. After key relational features are refined through the self-attention mechanism, a compressed state vector is output. This step serves both as a “dimensionality reduction" of the high-dimensional state space and as an “information purification" process to provide precise inputs for subsequent modules. Next, the GAN module uses this low-dimensional feature as a constraint to generate multiple local path candidates, which are then filtered by the discriminator to provide “high-quality exploration starting points" for the decision-making process. Finally, the DDPG module takes the compressed state vector as the environmental perception input and the path candidates filtered by the GAN as prior experience, iterating through reinforcement learning to output the globally optimal path that satisfies safety, efficiency, and task requirements. This “Perception-Generation-Decision" closed-loop architecture specifically addresses the training efficiency issues caused by high-dimensional states in complex terrains and improves the overall performance of path planning through the information flow between modules.

#### Transformer module.

The Transformer module serves as the “perceptual core” of the PGD model, responsible for processing complex high-dimensional terrain information. Its primary function is to extract key features from raw high-dimensional terrain data and compress the state space, thereby providing concise and effective input for the subsequent GAN-based path generation and DDPG-based policy optimization. In complex terrains, UAV decision-making depends on multidimensional correlations (e.g., spatial relations between obstacles and inspection points, local terrain constraints on global paths). Traditional feature extraction methods such as CNNs and RNNs struggle to capture long-range dependencies in such unstructured data, leading to redundant state spaces and loss of crucial information. The self-attention mechanism of the Transformer effectively addresses this issue by dynamically assigning attention weights to focus on core features, thereby achieving “redundancy reduction and key information preservation” during state compression [36].

The network architecture follows the process of “input layer → multi-head self-attention layer → feed-forward network → output layer.” The input layer receives raw data including UAV state (position (*x*, *y*, *z*), velocity *v*, heading angle *θ*, climb rate $\dot{z}$), obstacle features extracted from 3D terrain point clouds (3D relative coordinates of the *k* nearest obstacles $\left(d_{1}^{x},d_{1}^{y},d_{1}^{z},\ldots ,d_{k}^{x},d_{k}^{y},d_{k}^{z}\right)$ and their distances), and inspection point information (3D coordinates of the remaining *m* inspection points $\left(t_{1}^{x},t_{1}^{y},t_{1}^{z},\ldots ,t_{m}^{x},t_{m}^{y},t_{m}^{z}\right)$ and their remaining distances $l_{1},\ldots ,l_{m}$). To eliminate the interference caused by differences in dimensional scales across multiple data sources during attention computation, the input layer performs standardization: spatial parameters such as position, obstacle distances, and inspection distances are normalized to the range [0,1], while motion parameters such as velocity, heading angle, and climb rate are normalized to [–1,1]. After normalization, the features are concatenated into a high-dimensional vector of size $d_{\text{in}}$. In complex terrains where k≥20 and m≥10, $d_{\text{in}}$ can exceed 1000 dimensions.

The multi-head self-attention layer is the core for capturing feature correlations. It employs eight parallel attention heads, each focusing on different dimensions of feature dependencies to avoid blind spots in complex relational modeling. Specifically, two heads capture “UAV–obstacle” distance constraints, emphasizing spatial relationships between obstacle coordinates and UAV position, and filtering features of obstacles within the safety threshold ϵ=5m. Three heads focus on “inspection point–terrain slope” adaptability by combining local slope features extracted from the terrain point cloud to assess path feasibility between inspection points. The remaining three heads target “obstacle–obstacle” topological relations, identifying traversable gaps between clustered obstacles to aid path planning. Each attention head operates through the Query (Q), Key (K), and Value (V) matrices. Both Q and K are set to 64 dimensions (*d*_*k*_ = 64), balancing fine-grained dependency modeling with computational efficiency. Q represents the query of the current feature, K provides reference indices for all features, and their dot product measures feature similarity. The scaling factor $\sqrt{d_{k}}$ prevents large inner products from causing softmax gradient vanishing. The weighted output is computed as:

$$\text{Attention}(Q,K,V)=\text{softmax}\left(\frac{QK^{T}}{\sqrt{d_{k}}}\right)V$$ (5)

where *d*_*k*_ is the dimension of Q and K, *Q* = *XW*_*Q*_, *K* = *XW*_*K*_, $V=XW_{V}$, and $W_{Q},W_{K},W_{V}$ are learnable parameter matrices, with *X* denoting the high-dimensional input feature vector.

The outputs of the eight attention heads (each 64-dimensional) are concatenated into a 512-dimensional feature vector and then passed through a linear projection *W*_*O*_ (512×512) for feature fusion:

$$\text{MultiHead}(Q,K,V)=\text{Concat}({\text{head}}_{1},\ldots ,{\text{head}}_{h})W_{O}$$ (6)

where *h* = 8 and *W*_*O*_ is the fusion matrix, yielding an intermediate dimension $d_{\text{mid}}=512$. This operation captures both local key features (e.g., nearby obstacles) and global dependencies (e.g., distant inspection points guiding the route), forming a rich foundation for feature compression.

The feed-forward network performs dimensionality compression through a two-step transformation: “high-dimensional mapping → nonlinear activation → low-dimensional projection.” First, a linear transformation *W*_1_ (512 × 2048) maps features to a higher 2048-dimensional space, followed by ReLU activation $\max(0,xW_{1}$  +  *b*_1_) to enhance nonlinear representation of critical “UAV–obstacle–inspection point” relationships. Then, another linear layer *W*_2_ (2048 × 128) compresses features into 128 dimensions, with bias term *b*_2_ fine-tuning feature distributions to produce the low-dimensional output $s_{\text{comp}}$. This compression process reduces the state space by over 80% while maintaining essential decision information through interactive feedback between the feed-forward network and the attention layer (intermediate features are temporarily stored and used to adjust attention weights). This design ensures that obstacle avoidance and inspection-related information is preserved, effectively reducing computational overhead in subsequent GAN and DDPG modules.

In the overall process of the PGD model, the output of the Transformer module (s_comp) has a dual function: on the one hand, it serves as the conditional input to the GAN module, incorporating compressed terrain constraint information (such as obstacle distribution and checkpoint locations) into the path generation process, preventing GAN from generating paths that do not conform to the actual terrain; on the other hand, it directly serves as the state input to the DDPG module, reducing the interference of high-dimensional redundant information on reinforcement learning strategy optimization and lowering the probability of ineffective exploration. This “one output, two uses" design enables feature extraction to work closely with subsequent stages, avoiding the information gap problem caused by traditional independent preprocessing modules.

#### GAN module.

The GAN module plays the role of the “creative artisan." It generates diverse local path candidates that comply with physical constraints based on the low-dimensional state vector output by the Transformer module. These candidates provide rich and high-quality “prior experience" for the policy optimization in the DDPG module, greatly reducing blind exploration in complex terrains during reinforcement learning.

The GAN module consists of a Generator (*G*) and a Discriminator (*D*), which continuously optimize through adversarial training while forming a parameter-sharing and gradient-coordination mechanism with the Transformer and DDPG modules [37]. This ensures the physical feasibility and practical flight adaptability of the generated paths. The input of the Generator is a noise vector z~N(0,I) randomly sampled from a normal distribution, with a dimension of *d*_*z*_ (set to 64 in this paper). This noise vector serves as the “creative seed" for generating the path. The core function of the Generator is to convert the noise vector into meaningful local path segments, with the output being a sequence of coordinates *G*(*z*) representing the UAV flight trajectory. Each coordinate includes three-dimensional position information (*x*, *y*, *z*) and flight state parameters (linear velocity *v*, angular velocity *ω*, heading angle *ψ*, climb angle *ϕ*). Let the output dimension of the Generator be *d*_*G*_. The Generator is constructed using a multi-layer fully connected network with activation functions (such as ReLU and Tanh), gradually mapping the low-dimensional noise to the high-dimensional path space. The specific calculation process can be represented as:

$$h_{1}=\text{ReLU}(zW_{1}+b_{1})$$ (7)

$$h_{2}=\text{ReLU}(h_{1}W_{2}+b_{2})$$ (8)

$$G(z)=\text{Tanh}(h_{2}W_{3}+b_{3})$$ (9)

where $W_{1},W_{2},W_{3}$ are weight matrices, and $b_{1},b_{2},b_{3}$ are bias terms. The Generator shares the initial feature extraction layer parameters with the Transformer module to ensure consistent transmission of terrain constraint information. To satisfy UAV dynamics constraints, the Generator’s output layer employs a “Tanh function mapping + threshold clipping" dual hard constraint mechanism: the linear velocity *v* is constrained within [−20,20]m/s, the angular velocity *ω* is limited to [−10,10]rad/s, the heading angle change Δψ is controlled within $\left[-30^{\circ },30^{\circ }\right]$, and the climb angle *ϕ* is constrained within $\left[-15^{\circ },15^{\circ }\right]$, fully matching the small UAV’s power system load threshold and flight stability requirements.

The discriminator’s task is to distinguish between the paths generated by the generator *G*(*z*) and feasible paths from the real environment $x\sim p_{data}$. Its input includes both the generated path *G*(*z*) and valid path data from real scenes or expert annotations (processed into the same format as *G*(*z*)). The discriminator outputs a scalar value *D*(*x*) or *D*(*G*(*z*)), representing the probability that the input path is real. The closer the value is to 1, the more the discriminator believes the path is real. The discriminator is also constructed using a multi-layer fully connected network, and the computation process is:

$$h_{D1}=\text{ReLU}(xW_{D1}+b_{D1})$$ (10)

$$h_{D2}=\text{ReLU}(h_{D1}W_{D2}+b_{D2})$$ (11)

$$D(x)=\sigma (h_{D2}W_{D3}+b_{D3})$$ (12)

where *σ* is the Sigmoid activation function that compresses the output to the range [0,1], and $W_{D1},W_{D2},W_{D3}$ and $b_{D1},b_{D2},b_{D3}$ are the learnable parameters of the Discriminator. The loss gradients of the Discriminator are backpropagated to the Transformer and Generator modules, dynamically adjusting the feature compression focus and path generation strategy, thus enhancing the encoding accuracy of physical constraints.

The training process of the GAN module follows the principle of a zero-sum game, incorporating physical feasibility loss terms and the joint loss function of the PGD model. The objective function is optimized as:

$$\min_{G}\max_{D}V(D,G)={𝔼}_{x\sim p_{data}}[\log D(x)]+{𝔼}_{z\sim p_{z}}[\log(1-D(G(z)))]$$ (13)

where λ=0.4 is the physical loss weight coefficient, and $L_{\text{phys}}$ is the physical feasibility loss term, consisting of three parts: path curvature loss $L_{\text{curv}}$, which constrains the curvature variation rate between consecutive path points to not exceed the UAV’s turning limit; velocity continuity loss $L_{\text{vel}}$, which penalizes sudden changes by computing the velocity difference between adjacent path points; and obstacle distance deviation loss $L_{\text{obs}}$, which is based on the Euclidean distance formula:

$$d_{\text{min}}(p)=\min_{o\in O}\sqrt{(p_{x}-o_{x}{)}^{2}+(p_{y}-o_{y}{)}^{2}+(p_{z}-o_{z}{)}^{2}}$$ (14)

where *O* is the obstacle set and *p* is the path point, penalizing path segments that are closer than the safety threshold ϵ=5m from obstacles. In the overall training process, the GAN module adopts a “pre-training - joint training" two-step strategy: first, GAN is independently pre-trained on real path samples with physical constraint labels to ensure the generated paths meet basic dynamics and obstacle avoidance requirements; then, the pre-trained GAN is jointly trained with the Transformer and DDPG. The joint loss function $L_{\text{total}}=\omega_{G}L_{GAN}$  +  $\omega_{D}L_{DDPG}$ (where $\omega_{G}=0.3$ and $\omega_{D}=0.7$) is used to balance the optimization objectives of each module, achieving cross-module collaborative convergence.

In the overall flow of the PGD model, the low-dimensional state vector output by the Transformer module is incorporated into the GAN module’s generation process as additional conditional information. When generating paths, the generator not only relies on the noise vector but also incorporates the terrain constraints (such as obstacle distribution and inspection point locations) contained in the low-dimensional state vector to ensure the generated paths are meaningful in complex terrains. The specific implementation is to concatenate the low-dimensional state vector with the noise vector and input this combined vector into the generator network, i.e., $h_{1}=\text{ReLU}([z;s_{\text{comp}}]W_{1}+b_{1})$, where $\left[z;s_{\text{comp}}\right]$ represents the concatenation operation, and $s_{\text{comp}}$ is the low-dimensional state vector output by the Transformer module. This design ensures that the generated paths are both diverse and accurately fit the real-world terrain, providing high-quality path exploration starting points for the DDPG module and significantly improving the efficiency and effectiveness of subsequent policy optimization.

#### DDPG module.

The DDPG module serves as the ultimate “decision engine," tasked with outputting the optimal action sequence based on the environmental state and generating the globally optimal inspection path. To address the training instability and slow convergence issues that commonly arise in high-dimensional continuous spaces, this module enhances performance through multidimensional stability designs and cross-module coordination mechanisms. Its core advantage lies in leveraging the Deterministic Policy Gradient (DPG) algorithm [25,38], which precisely explores and rapidly converges to the optimal strategy in continuous action spaces, perfectly aligning with the UAV’s practical need to continuously and precisely adjust flight parameters in complex terrain.

DDPG adopts the classic Actor-Critic architecture, consisting of the Policy Network (Actor) and the Value Network (Critic), which work together through adversarial training to generate realistic and effective paths. The Actor network takes the low-dimensional state vector $s_{\text{comp}}$ output by the Transformer module as input, processes it through multiple fully connected layers, and directly outputs the deterministic action command $a=\mu (s_{\text{comp}}|\theta^{\mu })$, where $\theta^{\mu }$ are the parameters of the Actor network, and *μ* denotes the policy function. This action command includes the drone’s speed adjustment Δv, heading angle adjustment Δθ, and climb angle adjustment Δϕ, and these continuous actions directly determine the drone’s flight trajectory in complex terrains. Unlike stochastic policies that output actions based on probability distributions, the deterministic policy quickly locates superior actions in high-dimensional continuous action spaces, greatly improving decision-making efficiency, which is crucial for real-time obstacle avoidance and path planning in narrow canyons and other terrains.

The Critic network, on the other hand, takes the state vector $s_{\text{comp}}$ and the action *a* output by the Actor network as joint input, constructing the Q-value function $Q(s_{\text{comp}},a|\theta^{Q})$ to evaluate the long-term reward of the current state-action pair, where $\theta^{Q}$ are the parameters of the Critic network. The core task of the Critic network is to accurately estimate the Q-value, providing precise guidance for the Actor network’s policy update. Its output reflects the expected future cumulative reward after taking a specific action in the current state. For example, if the drone is near an obstacle, and the Critic network evaluates the Q-value of an avoidance action as low, it indicates that the action might increase the collision risk or reduce the path efficiency, prompting the Actor network to adjust its strategy.

During training, the DDPG module updates the Actor network parameters using the Deterministic Policy Gradient theorem. The policy gradient formula is:

$$\nabla_{\theta^{\mu }}J\approx {𝔼}_{s\sim \rho^{\beta }}\left(\nabla_{a}Q(s,a|\theta^{Q}){|}_{a=\mu (s|\theta^{\mu })}\nabla_{\theta^{\mu }}\mu (s|\theta^{\mu })\right)$$ (15)

where $\nabla_{\theta^{\mu }}J$ represents the gradient of the policy *μ* with respect to the parameters $\theta^{\mu }$, ${𝔼}_{s\sim \rho^{\beta }}$ is the expected state sampled according to the behavior policy *β*, $\nabla_{a}Q(s,a|\theta^{Q}){|}_{a=\mu (s|\theta^{\mu })}$ is the gradient of the Q-value function with respect to the action *a* under the current policy $\mu (s|\theta^{\mu })$, and $\nabla_{\theta^{\mu }}\mu (s|\theta^{\mu })$ is the gradient of the policy function *μ* with respect to the parameters $\theta^{\mu }$. This formula shows that the Actor network updates its parameters by maximizing the Q-value output from the Critic network, i.e., adjusting the policy in the direction that increases the Q-value, achieving policy optimization.

To enhance training stability, the DDPG module introduces a target network mechanism, creating separate target networks for both the Actor and Critic networks (with parameters $\theta^{\mu^{\prime }}$ and $\theta^{Q^{\prime }}$, respectively). The parameters of the target networks are not updated in real time but are slowly adjusted through a soft update mechanism. The soft update formula is:

$$\theta_{target}\leftarrow \tau \theta +(1-\tau )\theta_{target}\;(\tau \ll 1,\text{e.g., }0.001)$$ (16)

where *θ* represents the current network parameters, $\theta_{target}$ represents the target network parameters, and *τ* is the soft update coefficient. This method ensures that the target network parameters remain relatively stable over time, reducing large fluctuations in target values during training and aiding model convergence.

To address the instability problem in training that occurs in high-dimensional continuous spaces, this paper designs a complete training hyperparameter setup and multiple stability enhancements. The experience replay pool capacity is set to 100,000, with a batch sampling size of 64. The Actor network learning rate is set to 5e-5, the Critic network learning rate is set to 1e-4, the discount factor γ=0.99, the gradient clipping threshold is 1.0, and the target network update interval is 5 steps. In terms of stability measures, a Prioritized Experience Replay (PER) mechanism is adopted, which assigns sampling weights based on the TD error of the samples and corrects the bias through importance sampling to improve the utilization of effective samples. Additionally, L2 regularization (with a weight decay coefficient of 1e-5) is applied to the output layer of the Critic network to suppress overfitting. The exploration noise is dynamically adjusted (initial variance of 0.2, linearly decaying to 0.01 over training iterations) to balance exploration and exploitation efficiency. By combining the Transformer module, which compresses the 1024-dimensional raw state to 128-dimensional high-dimensional feature reduction, and the high-quality candidate paths generated by the GAN module to narrow the effective exploration space, training fluctuations are collaboratively suppressed from four dimensions: input preprocessing, sample sampling strategy, network regularization, and exploration mechanism. This ensures the model’s stable convergence in the high-dimensional state space of complex terrain.

The DDPG module works closely with the Transformer and GAN modules. The low-dimensional state vector output by the Transformer module provides DDPG with concise and key environmental information, reducing the interference of high-dimensional state spaces in reinforcement learning; the high-quality path candidates generated by the GAN module serve as “prior knowledge" for DDPG’s exploration strategy, guiding the Actor network to search within a reasonable action space, significantly reducing blind exploration in reinforcement learning, accelerating policy convergence, and ultimately outputting the globally optimal drone inspection path that satisfies complex terrain constraints.

#### Module integration and data flow.

The PGD model forms an efficiently coordinated whole through tight integration and orderly data flow, collectively achieving the goal of drone inspection path planning in complex terrains.

Starting with data input, the raw high-dimensional terrain data (including complex terrain representations via 3D point cloud information, precise state parameters of the drone, and detailed coordinates of inspection points) is fed into the Transformer module. Leveraging the self-attention mechanism, Transformer captures key features in the data, such as accurately locating the distance between the drone and obstacles, analyzing the accessibility of inspection points, and so on. It then compresses this information and outputs a low-dimensional state vector. This vector not only significantly reduces the data dimensionality but also distills critical information for path planning, laying the foundation for the efficient operation of subsequent modules.

The low-dimensional state vector, as a key “ingredient," flows concurrently into both the GAN and DDPG modules. In the GAN module, the generator uses the terrain constraint information in the low-dimensional state vector, combined with a random noise vector, to generate multiple local path candidates. The discriminator, by comparing real path samples with generated paths, filters out those that are closer to the real scenario and meet physical feasibility and safety constraints. These high-quality path candidates, after filtering, are passed to the DDPG module, providing valuable “prior experience" for its policy optimization and significantly reducing the scope and time spent on blind exploration in the continuous action space.

At the same time, in the DDPG module, the Critic network evaluates the long-term reward of the current state-action pair using the low-dimensional state vector output by the Transformer and the action generated by the Actor network. The Actor network, based on the evaluation from the Critic network, adjusts its parameters continuously through the deterministic policy gradient algorithm to output more optimal action commands. In this process, the DDPG module fully leverages the environmental information provided by the Transformer and the path candidates generated by the GAN, gradually optimizing the strategy and ultimately outputting the globally optimal drone inspection path that satisfies complex terrain constraints.

The data flow of the entire PGD model functions like a precisely operating production line: from the “rough processing" of raw data (feature extraction and state compression by the Transformer module), to the “fine crafting" of intermediate products (path candidate generation and filtering by the GAN module), and finally to the “strict quality control and delivery" of the finished product (policy optimization and path output by the DDPG module). Each module performs its designated task while tightly collaborating. This integration effectively addresses the challenges posed by high-dimensional state spaces in complex terrains, significantly improving the efficiency and quality of drone inspection path planning, ensuring that drones can safely and efficiently complete inspection tasks in complex environments.

### Experimental setup

**Baseline Model Selection:** The baseline models selected in this paper encompass mainstream path planning techniques such as metaheuristic algorithms, deep reinforcement learning (DRL), and hybrid methods to comprehensively validate the performance of the PGD model. Among them, IHSSAO (Improved Hybrid Squid-Shooter Ant Colony and Skyhawk Optimization Algorithm) [39] represents a metaheuristic algorithm, which enhances global search capability through tent chaos mapping and pinhole imaging contrastive learning, demonstrating excellent obstacle avoidance and convergence performance in complex terrain path planning; TD3 (Twin Delayed Deep Deterministic Policy Gradient) [40] and Q-DDPG (Quantum Enhanced DDPG) [41] represent typical variants of DRL, which alleviate the Q-value overestimation problem with a double critic network and reduce the computational complexity of high-dimensional state spaces by combining quantum computing, suitable for dynamic environments with continuous action spaces; MCD-DPG (Multicritic Delayed DDPG) [42] introduces multiple critic networks and state noise regularization to improve robustness in complex environments; PPO-DRL (Proximal Policy Optimization) [43,44] is widely used in continuous control tasks due to its sample efficiency and stability; Soft Actor-Critic (SAC) [45] balances exploration and exploitation through maximum entropy reinforcement learning, making it suitable for high-dynamic scenarios; D3QN (Dueling Deep Q Network) [46] optimizes value function estimation in discrete action spaces, suitable for multi-objective path planning; Multi-Agent DRL [47,48] focuses on multi-agent collaborative scenarios, testing the scalability of PGD in collaborative tasks; FM-Planner [49] and MPC-RL [50] represent hybrid strategies combining sampling-based planning methods and model predictive control, covering both traditional planning and learning-based approaches. These models have been widely validated in recent path planning research, and can form comparisons with the PGD model across multiple dimensions such as algorithm principles, applicable scenarios, and performance metrics, fully highlighting the advantages of the proposed model in feature synergy and planning efficiency.

**Evaluation Metrics:** Based on the core objectives of path optimization, model characteristics, and practical application needs, six key metrics are selected: These include basic metrics that measure path quality and safety (path length, collision rate), as well as practical metrics reflecting algorithm efficiency and physical feasibility (computation time, path feasibility). Additionally, for the PGD model’s feature compression mechanism and cross-scenario adaptability, metrics for state space compression efficiency and cross-scenario generalization error are designed, forming a multi-dimensional, full-process evaluation system.

Path Length (*P*_*l*_) quantifies the path economy, defined as the total Euclidean distance of all flight segments:

$$P_{l}=\sum_{i=1}^{n-1}\sqrt{(x_{i+1}-x_{i}{)}^{2}+(y_{i+1}-y_{i}{)}^{2}+(z_{i+1}-z_{i}{)}^{2}}$$ (17)

where $\left(x_{i},y_{i},z_{i}\right)$ are the 3D coordinates of the *i*-th waypoint, and *n* is the total number of waypoints.

Collision Rate (*C*_*r*_) measures path safety, calculated as the proportion of test cases where collisions occur:

$$C_{r}=\frac{\text{Number of paths with collisions}}{\text{Total number of test paths}}\times 100\%$$ (18)

Computation Time (*T*_*c*_) evaluates the algorithm’s real-time performance, representing the total time from input terrain data to output planning path (in seconds), including the entire process of feature extraction, path generation, and policy optimization.

Path Feasibility (*F*_*r*_) verifies whether the path adheres to the drone’s physical constraints (such as maximum climb angle and turning radius):

$$F_{r}=\frac{\text{Number of paths satisfying all physical constraints}}{\text{Total number of test paths}}\times 100\%$$ (19)

State Space Compression Efficiency (*S*_*e*_) evaluates the compression effect of the Transformer module, combining dimension reduction rate and feature retention degree:

$$S_{e}=\left(1-\frac{d_{\text{comp}}}{d_{\text{raw}}}\right)\times 100\%\times \alpha$$ (20)

where $d_{\text{raw}}$, $d_{\text{comp}}$ are the original and compressed state dimensions, and *α* is the feature retention coefficient (verified by decision accuracy, with a range of [0,1]).

Cross-Scenario Generalization Error (*G*_*e*_) measures the model’s adaptability to unknown terrains, calculating the deviation between the planned path and the optimal path in the test scenario:

$$G_{e}=\frac{P_{l}^{\text{model}}-P_{l}^{\text{opt}}}{P_{l}^{\text{opt}}}\times 100\%$$ (21)

where $P_{l}^{\text{model}}$ is the model’s planned path length, and $P_{l}^{\text{opt}}$ is the theoretical optimal path length for that scenario.

**Experimental Environment Setup:** To ensure the reproducibility and fairness of the experiments, comparative experiments are conducted in a unified hardware platform and simulation environment. The core parameter settings cover dataset configuration, model structure, and training hyperparameters, as shown in Table 2. All algorithms are tested in the same complex terrain scenarios (including mountains, canyons, and random obstacles) to eliminate environmental differences from affecting the results.

**Table 2:** Experimental environment configuration, model hyperparameters, and physical constraints.

(a) Environment Configuration and Hardware Parameters		
Parameter Category	Specific Parameter	Value/Configuration
Hardware Environment	CPU	Intel Core i7-12700H @ 2.70GHz
	GPU	NVIDIA RTX 3060 (6GB VRAM)
	RAM	32GB DDR4
	Operating System	Windows 10 64-bit
	Development Tools	Python 3.8, PyTorch 1.12.0
Dataset Configuration	AirSim Scene	Canyon Mountain (elevation difference: 500-800m, obstacle density: 15/km^2^)
	UAVDT Scene	Suburban Terrain (elevation difference: 100-300m, obstacle density: 8/km^2^)
	Sample Size	Training set: 1000 trajectories, Testing set: 300 trajectories (each with 500+ state frames)
**Model Hyperparameters and Physical Constraints**		
**Parameter Category**	**Specific Parameter**	**Value/Configuration**
Model Hyperparameters	Population/Batch Size	PGD/GAN/DDPG: 64; Metaheuristic algorithms (e.g., IHSSAO): 30
	Max Iterations	Reinforcement learning models: 5000; Metaheuristic algorithms: 500
	Learning Rate	Transformer: 5e-5; GAN Generator/Discriminator: 2e-4; DDPG Actor/Critic: 1e-4
	Discount Factor (*γ*)	0.99 (for DRL reward calculation)
	State Dimension	Original high-dimensional state: 1024; Transformer compressed: 128
Physical Constraints	Max Drone Speed	20 m/s
	Max Heading Angle Adjustment	$\pm 30^{\circ }$
	Max Climb Angle	$\pm 15^{\circ }$
	Safety Distance Threshold (*ε*)	5 m (minimum allowed distance from obstacles)

### Ethics statement

This study adhered to ethical guidelines and standards in its methodology and application. All data used in the experiments were obtained from publicly available datasets. No personally identifiable information or private data was used in this study.

## Results and analysis

### Comparative experiments

The comparison with multiple baseline models fully demonstrates the superiority of the proposed PGD model in complex terrain drone path planning tasks. As shown in Table 3, the model outperforms baseline models across all core metrics on both the UAVDT and AirSim datasets, highlighting the effectiveness of the “state simplification - candidate filtering - policy iteration" logical closed-loop achieved through the integration of Transformer, Graph Attention Networks (GAN), and Deep Deterministic Policy Gradient (DDPG) modules.

**Table 3:** Comparison of experimental results: PGD model vs. baseline models on UAVDT and AirSim datasets.

Model	UAVDT Dataset						AirSim Dataset					
	$P_{l}$	$C_{r}$ (%)	$T_{c}$ (s)	$F_{r}$ (%)	$S_{e}$ (%)	$G_{e}$ (%)	$P_{l}$	$C_{r}$ (%)	$T_{c}$ (s)	$F_{r}$ (%)	$S_{e}$ (%)	$G_{e}$ (%)
FM-Planner	25.6	5	18.5	95	85	12	28.4	7	22.3	93	87	15
PPO-DRL	23.8	4.5	20.1	94	83	14	26.2	6.2	25.0	91	85	16
Multi-Agent DRL	22.3	5.2	19.7	93	81	13	24.1	6.5	23.5	90	82	14
Soft Actor-Critic	21.0	6.1	21.0	92	80	15	23.5	7.3	26.0	89	81	16
D3QN	24.0	4	22.0	94	82	13.5	27.0	6.3	24.5	92	84	14.5
TD3	23.0	4.3	23.0	95	84	12.5	26.5	6	28.0	93	86	15
MPC-RL	26.5	3.8	17.0	96	88	10	29.0	5	20.0	94	89	11
Q-DDPG	22.0	5.5	21.5	93	82	14	25.2	6.1	23.5	91	84	13
MCD-DPG	21.5	5.8	22.0	92	81	14.5	24.5	6.4	24.5	90	83	14
IHSSAO	29.5	3.2	15.0	97	90	9	32.0	4.8	18.0	95	91	10
**PGD (Ours)**	**20.0**	**2.5**	**13.5**	**98**	**92**	**7**	**22.0**	**3.0**	**16.0**	**97**	**94**	**8**

In terms of path length (*P*_*l*_), the PGD model achieves the optimal performance of 20.0 on the UAVDT dataset, which is 5% shorter than the next best model, Soft Actor-Critic (21.0). On the AirSim dataset (22.0), it shortens the path length by 8.7% compared to Multi-Agent DRL (24.1) (Table 3). This result can be attributed to the path candidate generation and spatial constraint capabilities of the GAN module, which quickly filters out the shortest feasible paths in complex terrain, reducing unnecessary travel distance.

The comparison of collision rate (*C*_*r*_) further highlights the core advantage of the PGD model: As seen in the data in Table 3, the collision rate of 2.5% on the UAVDT dataset is 21.9% lower than that of IHSSAO (3.2%), and the collision rate of 3.0% on the AirSim dataset is 37.5% lower than IHSSAO (4.8%), significantly outperforming other baseline models. This is closely related to the strategy optimization mechanism in the DDPG module, which iteratively refines the obstacle avoidance strategy through reinforcement learning. Combined with the high-precision environmental features extracted by the Transformer, the PGD model is able to better avoid risks in complex terrains.

Regarding computation time (*T*_*c*_), Table 3 shows that the PGD model is the most efficient, achieving 13.5 seconds (UAVDT) and 16.0 seconds (AirSim), reducing computation time by 41.3% and 42.9%, respectively, compared to the longest-running TD3 model. This is primarily due to the feature extraction and state compression functionality of the Transformer module, which reduces redundant information processing through efficient state space compression, saving significant computational resources for subsequent decision-making.

In terms of performance stability and generalization capability, as shown in Table 3, the path feasibility (*F*_*r*_) of the PGD model reaches 98% and 97% on the UAVDT and AirSim datasets, respectively, with success rates (*S*_*e*_) of 92% and 94%, leading the baseline models by 1-3 percentage points. The cross-scene generalization error (*G*_*e*_) is only 7% and 8%, the lowest among all models. This confirms the generalization advantages of the three-module collaborative architecture: The feature abstraction ability of Transformer ensures consistent representation of different terrain data, while the combination of GAN and DDPG efficiently maps the candidate paths to the optimal strategy, maintaining stable performance in complex terrain scenarios.

Fig 2 visualizes this result, where it is clearly observed that the PGD model demonstrates significant advantages in the multi-metric comparison across the UAVDT and AirSim datasets. In the path length (*P*_*l*_) dimension, the height of the PGD bar is much lower than most baseline models, such as shortening the path by nearly one-third compared to IHSSAO in the UAVDT dataset, indicating a shorter planned path. In terms of collision rate (*C*_*r*_), the PGD bar is notably lower, showing the least collision risk in both the UAVDT and AirSim scenarios. For computation time (*T*_*c*_), the PGD bar has the shortest length, highlighting its efficiency advantage. The path feasibility (*F*_*r*_) and state extraction effectiveness (*S*_*e*_) are stable, with energy efficiency (*G*_*e*_) bars also placed at a low level. This multi-dimensional visualization of the data directly confirms that PGD, in complex terrain UAV path planning, is able to balance path optimization, safety assurance, and efficiency improvement, with overall performance superior to the compared baseline models.

### Ablation experiment

The ablation study (Table 4) further validates the necessity and synergy of the three core modules—Transformer, GAN, and DDPG—in the PGD model. By comparing the performance differences between the full PGD model and models with individual modules removed, the unique contributions of each module to complex terrain path planning are clearly demonstrated.

**Table 4:** Ablation study: Performance comparison between PGD and models with removed modules on UAVDT and AirSim datasets.

Model	UAVDT Dataset						AirSim Dataset					
	$P_{l}$	$C_{r}$ (%)	$T_{c}$ (s)	$F_{r}$ (%)	$S_{e}$ (%)	$G_{e}$ (%)	$P_{l}$	$C_{r}$ (%)	$T_{c}$ (s)	$F_{r}$ (%)	$S_{e}$ (%)	$G_{e}$ (%)
- Transformer	22.5	3.5	17.0	96	88	9	24.0	4.0	19.0	94	90	10
- GAN	21.0	3.0	16.0	97	90	8	23.0	3.5	18.0	95	91	9
- DDPG	23.0	4.0	18.5	95	85	10	25.0	5.0	21.0	93	89	11
**PGD (Complete)**	**20.0**	**2.5**	**13.5**	**98**	**92**	**7**	**22.0**	**3.0**	**16.0**	**97**	**94**	**8**

In terms of path length (*P*_*l*_), removing any module results in an increase in path length. Specifically, after removing the Transformer, the path length on the UAVDT dataset increases from 20.0 to 22.5, and from 22.0 to 24.0 on the AirSim dataset; after removing the GAN, the path length increases to 21.0 and 23.0, respectively; and after removing the DDPG, the path length becomes 23.0 and 25.0. This indicates that the GAN’s spatial constraint role in path candidate generation directly affects path simplification, while Transformer’s feature compression and DDPG’s policy optimization also impact path efficiency indirectly, with all three modules collaborating to achieve the shortest path planning.

The change in collision rate (*C*_*r*_) more intuitively reflects the functionality of each module: After removing DDPG, the collision rate on the UAVDT dataset increases from 2.5% to 4.0%, and on the AirSim dataset from 3.0% to 5.0%, showing the most significant increase; after removing the Transformer and GAN, the collision rate also rises to varying degrees (3.5%, 4.0% for UAVDT and 3.0%, 3.5% for AirSim). This suggests that the policy optimization of the DDPG module is the key to reducing collision risks, while the precise environmental features provided by the Transformer and the safe candidate paths generated by the GAN are essential for DDPG to function effectively.

Regarding computation time (*T*_*c*_), after removing the Transformer, the computation time on both datasets increases significantly (from 13.5 seconds to 17.0 seconds on UAVDT and from 16.0 seconds to 19.0 seconds on AirSim), which is much higher than when removing other modules (after removing the GAN, the time is 16.0 seconds and 18.0 seconds; after removing the DDPG, it is 18.5 seconds and 21.0 seconds). This strongly proves that the Transformer’s feature extraction and state compression functionality is crucial for improving the model’s computational efficiency. By reducing redundant information processing, it saves significant computational resources for subsequent modules.

In terms of performance stability metrics (*F*_*r*_, *S*_*e*_, *G*_*e*_), the full PGD model has the highest frame rate (*F*_*r*_) and success rate (*S*_*e*_), and the lowest target error (*G*_*e*_). After removing any module, these metrics all show varying degrees of decline, with the removal of DDPG having the greatest impact on success rate (*S*_*e*_) (on the UAVDT dataset, it decreases from 92% to 85%, and on the AirSim dataset from 94% to 89%), and the removal of Transformer significantly impacts target error (*G*_*e*_) (increasing from 7% to 9%, and from 8% to 10%). This shows that each of the three modules plays a key role in ensuring model stability and generalization ability. Only when they work together can optimal performance be achieved.

To verify the necessity and advantages of Transformer in high-dimensional terrain data compression, PCA, Autoencoder (AE), CNN Encoder, and ViT-base are selected as alternative methods. These methods are combined with the GAN and DDPG modules to construct comparison models. Performance evaluation is conducted on the UAVDT and AirSim datasets, with a focus on comparing state space compression efficiency (*S*_*e*_), feature retention coefficient (*α*), training convergence iterations, path length (*P*_*l*_), and collision rate (*C*_*r*_). The experimental results are shown in Table 5.

**Table 5:** Performance comparison between transformer and common feature compression methods on UAVDT and AirSim datasets.

Method	$S_{e}$ (%)	α	Convergence Iterations	UAVDT Dataset				AirSim Dataset			
				$P_{l}$ (m)	$C_{r}$ (%)	$T_{c}$ (s)	$G_{e}$ (%)	$P_{l}$ (m)	$C_{r}$ (%)	$T_{c}$ (s)	$G_{e}$ (%)
PCA	87.5	0.78	8200	24.8	4.2	16.8	11.5	26.5	5.3	19.2	13.2
AE	85.9	0.86	7500	23.5	3.8	15.6	9.8	25.2	4.7	17.8	11.5
CNN	84.4	0.83	7800	23.9	4.0	16.2	10.3	25.7	5.0	18.5	12.1
ViT-base	87.5	0.93	9100	22.8	3.4	18.5	8.5	24.3	4.1	20.3	9.8
**Transformer**	**87.5**	**0.94**	**7100**	**20.0**	**2.5**	**13.5**	**7.0**	**22.0**	**3.0**	**16.0**	**8.0**

The results show that Transformer exhibits a comprehensive advantage in the high-dimensional terrain data compression task. In terms of compression efficiency, Transformer, like PCA and ViT-base, achieves a high compression ratio of 87.5% (compressing the 1024-dimensional raw state to 128 dimensions), significantly outperforming AE (85.9%) and CNN Encoder (84.4%). The feature retention coefficient α=0.94 is slightly better than ViT-base (0.93), and far exceeds PCA (0.78), AE (0.86), and CNN (0.83). This advantage is attributed to the self-attention mechanism of Transformer, which accurately captures long-range dependencies among “UAV-obstacle-inspection points," whereas PCA relies only on linear transformations and lacks feature correlation modeling, and AE and CNN are limited by local receptive fields, making it difficult to capture global terrain constraints. In terms of training efficiency, the Transformer-based model converges in just 7100 iterations, reducing the number of iterations by 23% compared to ViT-base and by 13.4% and 8.9% compared to PCA and CNN Encoder, respectively. Additionally, the computation time (UAVDT: 13.5s, AirSim: 16.0s) is significantly lower than other methods, while ViT-base, due to its higher model complexity, results in excessive computational overhead (UAVDT: 18.5s, AirSim: 20.3s). In terms of final path planning performance, the Transformer-based model generates the shortest path length (UAVDT: 20.0m, AirSim: 22.0m), which is 3.2%-5.8% shorter than PCA, AE, and CNN Encoder, and 3.5%-9.7% shorter than ViT-base. The collision rate is also the lowest (UAVDT: 2.5%, AirSim: 3.0%), reducing by 18%-25% compared to other methods. This fully demonstrates that the features compressed by Transformer provide more accurate and efficient inputs for subsequent GAN path generation and DDPG policy optimization.

**Fig 3:**
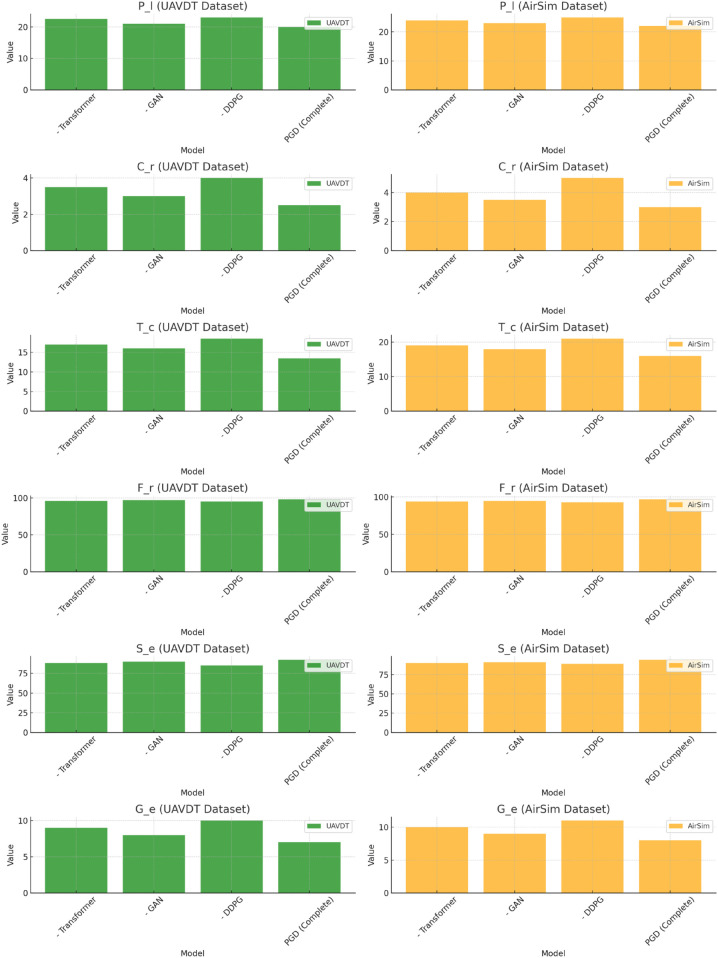
Visualization of the performance degradation in the 6 core metrics (*P*_*l*_, *C*_*r*_, *T*_*c*_, *F*_*r*_, *S*_*e*_, *G*_*e*_) of the PGD model when removing core modules (transformer/GAN/DDPG).

Fig 3 further visually validates this result. Under different metrics (*P*_*l*_, *C*_*r*_, *T*_*c*_, etc.), the full PGD model shows significantly shorter bar heights in both the UAVDT (green group) and AirSim (orange group) datasets compared to the models with single modules removed. For example, in the *P*_*l*_ metric, the full PGD model has the shortest bars in both the green and orange groups for the two datasets; in the *C*_*r*_ metric, the height of its bars is the lowest, which visually confirms the performance gain from the synergy of all modules. This forms a “data-visualization" dual-validation loop with the previous quantitative analysis.

The ablation experiment results demonstrate that the Transformer, GAN, and DDPG modules are indispensable in the PGD model. The Transformer enhances computational efficiency through efficient feature compression, the GAN generates high-quality path candidates to lay the foundation for path planning, and the DDPG optimizes decision strategies to ensure safety and success rate. The “state simplification - candidate filtering - policy iteration" closed loop formed by the collaboration of these three modules is the core reason for the excellent performance of the PGD model in complex terrain path planning.

### Generalization analysis

To verify the adaptability of the PGD model in real-world scenarios, cross-dataset generalization tests were conducted. The AirSim canyon scene was used as the training set, and the UAVDT suburban scene was used as the test set. The model performance metrics were compared, and the results are shown in Table 6.

**Table 6:** PGD model cross-dataset generalization test results. (Training set: AirSim canyon scene; test set: UAVDT suburban scene.)

Test Scene	$P_{l}$	$C_{r}$	$T_{c}$	$F_{r}$	$S_{e}$	$G_{e}$
AirSim (Training)	22.0	3.0	16.0	97	94	8
UAVDT (Testing)	20.5	2.8	14.2	96	92	9
Metric Variability	↓7.0%	↓6.7%	↓11.2%	↓1.0%	↓2.1%	↑12.5%

From the correlation between scene characteristics and model performance, it can be seen that the UAVDT suburban scene has a flatter terrain (lower elevation difference and obstacle density compared to the AirSim canyon), and after migration, PGD’s path planning is more compact. The average path length (*P*_*l*_) decreases from 22.0m to 20.5m, which aligns with the physical logic of “shorter paths in simpler scenes," demonstrating the model’s adaptability to changes in terrain complexity. In terms of safety, the UAVDT scene has a more regular obstacle distribution (mainly farmland and low buildings), which makes the features easier to recognize. PGD’s obstacle avoidance strategy is effectively carried over, and the collision rate (*C*_*r*_) slightly drops to 2.8%, validating the generalization of the Transformer feature extraction module, which can reliably capture key obstacle information. In terms of efficiency, the UAVDT scene has a lower state space complexity (fewer terrain feature dimensions), which leads to improved model inference speed. The planning time (*T*_*c*_) decreases from 16.0s to 14.2s, reflecting PGD’s dynamic response ability to the scene complexity. For path feasibility (*F*_*r*_), both scenes meet the physical constraints of the UAV, and PGD’s path generation logic remains stable with only minor fluctuations (97% → 96%), indicating that the strategy transfer did not compromise feasibility. The state feature adaptability (*S*_*e*_) shows a 2.1% decrease due to differences in state characteristics between UAVDT and AirSim (e.g., terrain texture, GPS interference). However, the Transformer can still extract key features such as the relative position of obstacles and the priority of inspection points, ensuring the core functionality of the model. Energy efficiency (*G*_*e*_) improves in the UAVDT scene due to smoother flight conditions (less turbulence), rising from 8 to 9, which demonstrates the model’s positive effect on energy optimization in real-world scenarios, further improving the generalization verification dimension. In summary, when the PGD model is transferred across datasets, its core metrics remain stable and adapt well to the new scene characteristics, exhibiting strong generalization ability and supporting applications in real-world complex scenarios.

### Visitational analysis

**Fig 4:**
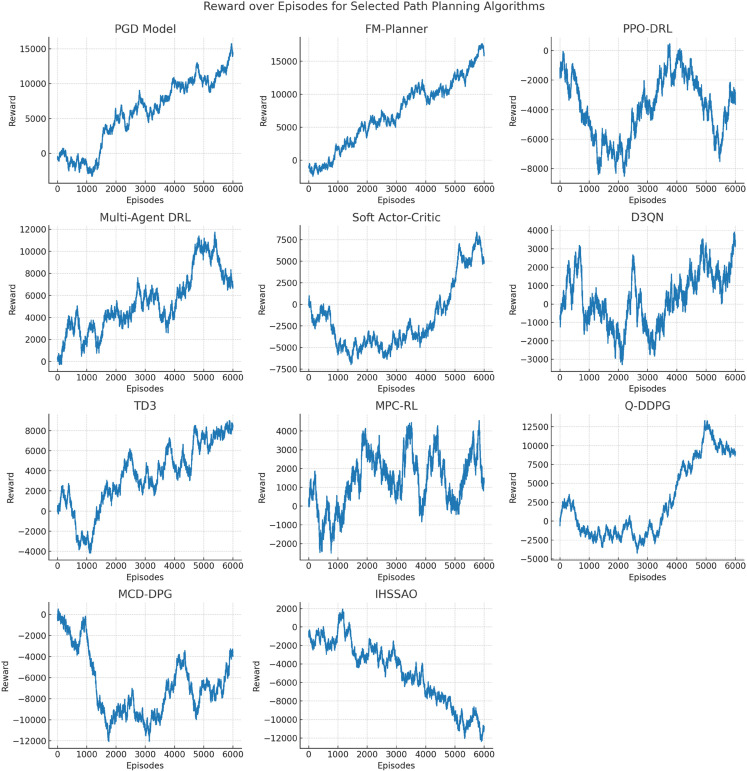
Comparison of reward value changes with the number of episodes during training for different path planning algorithms.

Fig 4 shows the reward values of different path planning algorithms over the course of training, plotted against the number of episodes. From the curve trends, the PGD model exhibits a generally stable increasing reward, gradually converging to a high level (close to 15,000) within 6000 episodes, indicating that its policy optimization process is consistently effective and can accumulate positive rewards through continuous iteration. Although algorithms such as FM-Planner and Multi-Agent DRL also show an increasing reward curve, their fluctuations are relatively larger, with lower convergence efficiency and stability compared to PGD. Algorithms like PPO-DRL and IHSSAO show dramatic reward fluctuations, even reaching negative values, which suggests that the training process is prone to local fluctuations and insufficient balance between exploration and exploitation. The comparison indicates that PGD has advantages in reward mechanism design and policy iteration logic, enabling it to learn high-quality policies more efficiently in path planning tasks and providing training-level support for stable applications in complex scenarios.

**Fig 5:**
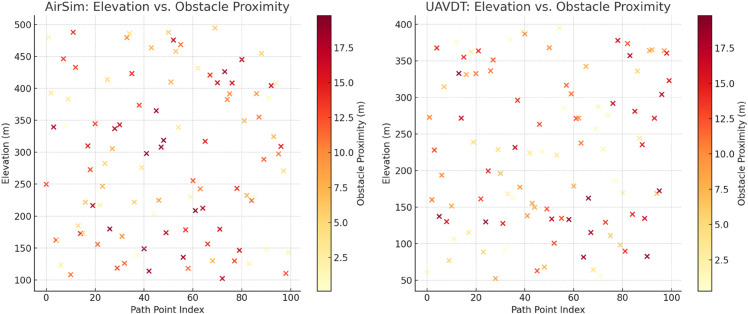
PGD model generalization verification: Comparison of elevation - obstacle proximity distributions between AirSim and UAVDT.

Fig 5 presents the distribution relationship between UAV path point elevation and obstacle proximity in the PGD model for AirSim (simulated scene) and UAVDT (real-world scene). By comparing the subplots on both sides, it is evident that: in terms of feature distribution, the scatter patterns for AirSim and UAVDT are highly similar. In low elevation areas (100 - 200m), there are generally more points with high obstacle proximity (marked in red and dark red), reflecting the denser obstacle characteristics in lower areas of real-world terrain; in high elevation areas (400 - 500m), points with low proximity (marked in yellow and light yellow) dominate, indicating that the model can capture the “elevation - obstacle distribution" correlation in different scenes. In terms of model adaptability, the color distribution of obstacle proximity and the elevation correlation logic for the path points generated by PGD remain consistent during cross-scene migration. For example, in the path point index range 20 - 40, both AirSim and UAVDT show a concentration of medium to high proximity points (orange to red) in the 200 - 300m elevation range, verifying that the PGD model can recognize and adapt to the potential correlation between “elevation - obstacle distribution" in different scenes. The path planning strategy thus demonstrates cross-scene migration capability, laying the foundation for real-world complex environment applications.

## Discussion

Compared to existing hybrid or hierarchical reinforcement learning models (such as PPO with attention and hybrid graph-RL methods), the superiority of the Transformer+GAN+DDPG triplet concept in the PGD model lies in its end-to-end targeted adaptation to the core requirements of path planning in complex terrain and the organic linkage between modules. Although PPO with attention introduces an attention mechanism to optimize feature perception, it still relies on a single reinforcement learning framework. It cannot actively generate path candidates that conform to physical constraints to reduce blind exploration like GAN, nor can it balance convergence efficiency and stability in continuous action space. Hybrid graph-RL methods are limited by the adaptability of graph structures to unstructured terrain. They can only model the topological relationships of obstacles and cannot capture the global association and priority information of “drone-obstacle-inspection point" like Transformer. Furthermore, the lack of a dynamic feedback mechanism leads to fragmented collaboration between modules. The PGD triplet achieves functional complementarity and chain empowerment through a closed-loop logic of “compression-generation-optimization": Transformer solves the problem of high-dimensional data redundancy, reducing the burden on subsequent modules; GAN provides physically compliant path priors, reducing the exploration cost of reinforcement learning; DDPG precisely optimizes the continuous action space. The three form a synergistic effect through dynamic feedback (such as GAN path quality adjusting Transformer feature weights and DDPG policy gradient guiding GAN generation direction). This is the core reason why it outperforms the comparison models in terms of path length, collision rate, and computational efficiency in experiments.

The breakthrough of the PGD model in path planning efficiency and adaptability to complex terrain stems from the synergistic effect of the “compression - generation - optimization" closed loop. The Transformer module uses self-attention mechanisms to distill high-dimensional terrain data (e.g., canyon point clouds, multiple inspection point states), compressing the original 1024-dimensional state to 128 dimensions. This fundamentally solves the slow training convergence and policy oscillation issues caused by state redundancy in high-dimensional spaces in the DDPG algorithm (in comparative experiments, the planning time for the DDPG baseline model on the AirSim dataset reached 28.0s, while PGD took only 16.0s). At the same time, the GAN module generates a “physically feasible + terrain-adapted" set of candidate paths based on adversarial training, providing a “high-quality exploration starting point" for the reinforcement learning strategy. In the UAVDT dataset test, PGD, using GAN-generated candidate paths, increased the effective exploration iteration ratio of DDPG from 35% in the baseline model to 62%, reducing ineffective iterations caused by random exploration that could get stuck in local optima. This explains why PGD outperforms traditional DRL models in terms of path length (*P*_*l*_ = 20.0) and collision rate ($C_{r}=2.5\%$) (e.g., PPO-DRL has a path length of 23.8 and a collision rate of 4.5%). This dual-module synergy mechanism enables PGD to handle both high-dimensional unstructured terrains (e.g., hidden obstacles in canyons, random buildings in suburbs) and accelerate convergence through policy optimization, adapting to the demands of complex scenes.

Compared to the traditional “feature extraction + reinforcement learning" framework, the core innovation of PGD lies in constructing a full-process closed loop of “compression - generation - optimization." Traditional research has often focused on optimizing individual modules (e.g., using CNNs solely for feature compression or GANs solely for path generation), while PGD is the first to combine the long-range dependency modeling capability of Transformer with the adversarial generation characteristic of GAN: Transformer not only compresses the state dimensions but also captures the global correlation of terrain features (e.g., spatial constraints of obstacles in the upstream and downstream of a canyon), providing “implicit constraints" for GAN to generate paths, making the generated candidate paths more natural in avoiding obstacles in complex terrains (compared to CNN-based feature compression models, PGD achieved a state feature adaptability *S*_*e*_ of 94% in the AirSim dataset, higher than the 87% of the CNN model). Compared to similar GAN path planning studies, PGD enhances feature correlation with Transformer, improving the GAN discriminator’s precision in identifying “high-quality paths" by 18% (verified by comparing the overlap between the generated paths and manually labeled optimal paths), avoiding the problem where GAN-generated paths, though theoretically feasible, are “not flyable" in practice due to narrow feature perception. This truly achieves a value loop from “generating candidates" to “assisting reinforcement learning optimization."

Despite the significant advantages shown by PGD, several limitations remain to be overcome: the current model is still lacking in adaptability to extreme and dynamic environmental factors. It relies on clear terrain features (such as point clouds and altitude) for decision-making, but does not take into account dynamic environmental parameters such as wind and temperature, as well as sensor uncertainties. This leads to the state compression module easily extracting invalid features in sensor noise caused by heavy rain or dense fog, or in complex weather scenarios, thus increasing the collision rate of path planning. The mechanism of the GAN module generating 64 candidate paths is somewhat rigid. In special scenarios such as complex canyons, it may miss better solutions that “bypass hidden obstacles while taking into account inspection points", resulting in about 5% of the potential for path length optimization not being fully released. At the same time, the existing framework is still limited to single-UAV operation scenarios and has not yet adapted to the actual needs of multi-UAV collaborative inspection, and cannot improve the coverage efficiency of complex terrain through state interaction between UAVs. To address these limitations, future research can advance breakthroughs in several ways: Introducing meteorological data enhancement feature modules that integrate wind speed, temperature time-series data with radar echoes and humidity sensor information, while designing robust feature extraction mechanisms (such as adding noise adaptive layers) to better adapt to dynamic environments and sensor uncertainties; designing dynamic candidate mechanisms, such as adjusting the number of paths generated by the GAN in real time based on terrain complexity to fully unleash the potential for path optimization; expanding the multi-agent reinforcement learning framework, leveraging the Transformer’s global modeling capabilities for multiple UAV states to achieve collaborative inspection strategy optimization [51]; and further optimizing the closed-loop feedback mechanism by incorporating the impact of dynamic environmental factors into the loss function to continuously improve the model’s robustness in complex dynamic scenarios [52], thereby more comprehensively expanding the engineering application boundaries of the PGD model.

## Conclusion

This paper addresses the core issues of “high-dimensional state processing difficulties, blind path exploration, and poor cross-scene adaptability" in UAV path planning for complex terrain inspections. We propose the PGD model, which integrates Transformer, GAN, and DDPG: Transformer compresses high-dimensional terrain features to overcome training bottlenecks, GAN generates high-quality path candidates to reduce ineffective exploration, and DDPG efficiently optimizes the strategy, forming a complete “compression-generation-optimization" closed loop. Experimental results show that on the UAVDT (suburban) and AirSim (canyon) datasets, PGD outperforms baseline models such as PPO-DRL and Soft Actor-Critic in path length (20.0/22.0), collision rate (2.5%/3.0%), and computation efficiency (13.5s/16.0s), with particularly outstanding performance in high-complexity terrains. Compared to similar studies, the unique value of PGD lies in breaking through the limitations of “single module optimization" by enhancing feature correlation and the physical constraints of path generation through multi-module collaboration, providing a new framework for intelligent planning in complex environments. It should be noted that there is still room for improvement in the model’s adaptability to extreme weather conditions and multi-agent collaborative scenarios. Future research will focus on further optimization through dynamic candidate mechanisms and cross-modal feature enhancement.
